# Supplementary material for: First estimates of inequality benchmark incomes for a range of countries
Source: PLoS One. 2021 Mar 17;16(3):e0248178. doi: 10.1371/journal.pone.0248178 (PMC7968638; doi:10.1371/journal.pone.0248178)
Supplement: S1 Table — (DOCX) [file pone.0248178.s001.docx]

**S1 Table. Inequality and benchmark percentiles in 2010 under assumption of equal incomes within deciles.**

|  | Inequality | | | | | Benchmark percentiles | | | | |
| --- | --- | --- | --- | --- | --- | --- | --- | --- | --- | --- |
|  | $I_{G}$ | $I_{MLD}$ | $I_{AG}$ | $I_{V}$ | $I_{K}$ | $p_{G}$ | $p_{MLD}$ | $p_{AG}$ | $p_{V}$ | $p_{K}$ |
| *Nordic* |  |  |  |  |  |  |  |  |  |  |
| Norway | 0.231 | 0.092 | 10,781 | 404.936 | 8,657 | 61.5 | 60.5 | 50 | 60.5 | 70.5 |
| Sweden | 0.236 | 0.097 | 8,067 | 218.533 | 6,404 | 61.8 | 60.5 | 50 | 60.5 | 70.5 |
| Denmark | 0.263 | 0.149 | 8,512 | 246.125 | 7,601 | 63.1 | 50.5 | 50 | 50.5 | 70.5 |
| *Anglo-Saxon* |  |  |  |  |  |  |  |  |  |  |
| UK | 0.321 | 0.174 | 10,534 | 420.232 | 12,817 | 66.1 | 60.5 | 50 | 60.5 | 70.5 |
| Ireland | 0.325 | 0.177 | 11,948 | 549.101 | 14,927 | 66.2 | 60.5 | 50 | 60.5 | 80.5 |
| USA | 0.400 | 0.300 | 17,600 | 1170.133 | 26,625 | 70.0 | 60.5 | 50 | 60.5 | 80.5 |
| *BRICS* |  |  |  |  |  |  |  |  |  |  |
| Russia | 0.369 | 0.234 | 5,252 | 105.426 | 7,410 | 68.5 | 60.5 | 50 | 60.5 | 80.5 |
| India | 0.407 | 0.275 | 1,251 | 7.338 | 2,388 | 70.4 | 70.5 | 50 | 70.5 | 80.5 |
| Brazil | 0.520 | 0.501 | 5,253 | 137.419 | 13,615 | 76.0 | 70.5 | 50 | 70.5 | 90.5 |
| S. Africa | 0.672 | 0.954 | 6,392 | 248.048 | 26,065 | 83.6 | 80.5 | 50 | 80.5 | 90.5 |

NOTES: 1. Source: WIID / Author’s calculations; 2. $I_{V}$ is expressed in millions; 3. Benchmark percentiles $p_{MLD}$ , $p_{V}$ and $p_{K}$ are based on the limits of the corresponding benchmark incomes as $\varepsilon\to0$.
