# Supplementary material for: First estimates of inequality benchmark incomes for a range of countries
Source: PLoS One. 2021 Mar 17;16(3):e0248178. doi: 10.1371/journal.pone.0248178 (PMC7968638; doi:10.1371/journal.pone.0248178)
Supplement: Attachment — Submitted filename: Response to Reviewers.docx [file pone.0248178.s003.docx]

Dear Professor Suen,

I am most grateful to you for allowing me the opportunity to submit a revised version of this paper. Both your colleague and the reviewer have provided some excellent suggestions and I believe the revised paper has improved in response to these. I have replied below, in bold italics, to each of these suggestions.

Please note that I have also corrected an error I spotted in the formula for the Absolute Gini’s benchmark income – I had estimated these benchmark incomes correctly, but the formula in the text was misleading.

I have tracked changes in the revised manuscript and also provided a clean, untracked version. Line numbers given throughout the response refer to the version with tracked changes.

I hope that the changes are acceptable to both you and the reviewers.

Kind regards,

Laurence Roope

Suggestions provide by Editor’s colleague:

1. The author may find this book useful / interesting: Deaton's *The Analysis of Household Surveys* (Ch. 3)

***RESPONSE: I am grateful to the reviewer for this suggestion. This chapter in Deaton’s book is an excellent resource.***

2. Some thoughts:

- would benchmark incomes *also* signify the *poorest* person whom it is just and fair *to tax*? This might be interesting to a broader range of public economists and policymakers!

***RESPONSE: I am grateful to the reviewer for this suggestion. I agree that an interpretation along these lines could be very interesting to a broad range of public economists and policymakers. Corvalan (2014) and Lambert (2014) discussed the interpretation of the benchmark as signifying the richest person in society for whom it is just and fair to subsidize their income. I did not go into much detail on this in the original manuscript, but have added more details in the revision.***

***In the case of relative inequality measures, this interpretation is based on subsidies financed via a proportional tax. A proportional tax leaves relative inequality unchanged. Moreover, in the case of the Gini coefficient, the person at the benchmark income remains the same following a proportional tax (though their income changes of course). Thus, the benchmark income can be used to identify the richest person for whom it might be deemed fair to subsidize income financed by a proportional tax. In the case of absolute measures such as the Absolute Gini, a similar argument can be made regarding subsidies financed by a lump sum tax – which leaves absolute inequality unchanged.***

***As these interpretations are based on taxing everyone prior to the subsequent subsidisation of certain incomes, we cannot cleanly describe benchmark incomes as “signifying the poorest person whom it is just and fair to tax.” However, we can certainly describe it as signifying the poorest person whom it is just and fair not to subsidise their income from a proportional tax (in the case of the Gini). I have added a discussion of these points in the revised manuscript in lines 68-74.***

- inequality and social welfare are not (necessarily) the same thing. It would be good to explain what benchmark incomes have to do with various desirable properties of social welfare functions, or with the canonical social welfare functions.

***RESPONSE: I am grateful to the reviewer for raising this point. As the reviewer rightly says, inequality and social welfare are not necessarily the same thing. As shown by Roope (2019), the key ingredient in a social preference relation that is needed to guarantee the existence of a benchmark income/position is simply a strong version of the Pigou–Dalton transfer property. Since this property underpins virtually all known inequality indices, so too the existence of benchmark incomes must be implied by virtually all inequality indices. As Roope (2019) discusses, this result can also be interpreted as indicating that the essence of a preference for the existence of a benchmark income/position is a strong concern for inequality.***

***In contrast to both social welfare functions and poverty measures, both of which typically satisfy a weak monotonicity property (where adding an increment to an individual’s income can never reduce social welfare or increase poverty), adding increments of income to those above benchmark incomes actually increases inequality, capturing the intuition that there is something socially undesirable about inequality.***

***I have added some text discussing these points in lines 41-47, explaining that a benchmark income/position is inconsistent with weak monotonicity, which is a standard assumption in social welfare functions.***

- poverty lines come in for a lot of criticism that could also apply to benchmark incomes. The argument would be strengthened by explaining why benchmark incomes are preferable.

***RESPONSE: I am very grateful to the reviewer for this suggestion, as it provides an opportunity to be clearer on what I think is a major strength of the benchmark income approach.***

***A major criticism of poverty lines is that they are largely arbitrary. There is no good theoretical basis for choosing a particular poverty line. This applies to relative poverty lines based on, for example, a certain percentage of the median income. It also applies to absolute poverty lines, such as those based on minimum baskets of goods, where all sorts of judgements must be made, such as what sorts of goods to include in a basket, how to make these comparable across countries and so on.***

***In contrast, an attractive feature of the benchmark income approach is that, for a given income distribution, it arises naturally and is uniquely determined by the social preferences (including the strong transfer principle) embodied in a given inequality measure. Thus, if one has already decided to use an inequality measure, and implicitly adopted its normative underpinnings, one must also accept the benchmark income that is implied by these underpinnings.***

***In the revised manuscript, I have outlined this advantage in a new paragraph (lines 49-59).***

- is the author looking at household or individual incomes? Given differences in labor sharing within households across countries, the ranking of countries could depend on the reporting unit.

***RESPONSE: The reviewer has raised a good point here. Where possible I tried to use individual incomes. This was the case for all countries apart from India and Russia. However, the data for India are based on household income, while unfortunately it is unclear in the WIID dataset whether Russia is household or individual. In the revised manuscript I have added text to the data section describing this clearly (lines 130-133).***

Reviewer #1: The author adopted smoothing algorithm proposed by Shorrocks and Wan (2008) to compute various inequality measures and benchmark incomes for 10 countries and their correlations between inequality measures and benchmark incomes using WIID 2010 data. The findings suggest the benchmark incomes lie far above the poverty line and therefore imply that any policies enhancing incomes for those with incomes between these two numbers cannot reduce poverty.
The focus on the paper is more on the quantitative side rather than the policy side. I have the following suggestions regarding the data and discussion:
1. Can the author elaborate more about the choice of 2010 data and the 10 countries?

***Thank-you for this suggestion. The analysis in this study was initially conducted some years ago. At this time, version 3.0b was the most recent version of the WIID, and 2010 was the most recent year with available data for most countries. The purpose of this short paper is to provide an empirical illustration of the benchmark income approach, for a selection of countries and using a range of inequality measures. As there are hardly any estimates of benchmark incomes in the literature, I wanted to include a diverse selection of countries that differed in terms of level of development, geographic region and inequality levels. Thus, for high-income country examples, I selected several countries that follow what might be termed an ‘Anglo-Saxon’ model, with a small welfare state and relatively high inequality levels; as well as several examples of a ‘Nordic’ model with a larger welfare state and relatively low inequality levels. As a contrast to both Anglo Saxon and Nordic countries, I included several ‘BRICS’ countries, which are notable due to both their increasing role in the global economy and often high levels of inequality.***

***I have expanded the data section substantially to elaborate on these choices (see lines 113-128).***

2. How much can we gain by using smoothing algorithm over constant-income-within-decile approach? Can the author provide more details about their differences or even generate the rankings using the latter approach?

***Smoothing approaches such as the Shorrocks and Wan (2008) alogorithm are widely regarded as preferable to assuming constant incomes within deciles. This is because the resulting smoothed distributions are more plausible, and assuming equal incomes within quantiles biases inequality estimates downwards (e.g. Anand and Segal 2008, Niño‐Zarazúa et al, 2017).***

***In the case of this study, as an indication of the differences, I have recomputed all the inequality and benchmark estimates in Table 2 and provided them in Supplementary Appendix Table S1 as a sensitivity analysis. As expected, all inequality estimates are lower. However, the rankings of countries according to each inequality measure are qualitatively similar. The rankings obtained by ordering countries by their benchmark percentiles are also similar. Note however that, with regard to estimating the percentiles in which benchmark incomes lie, a limitation of this assumption is that, for most of the inequality measures, it makes it impossible to locate where benchmark incomes lie within a particular decile. Instead, estimates will typically lie between two deciles and it will not be possible to identify, for example, whether they lie in, say, the 62nd, 65th or 68th percentile. The one exception is the Gini coefficient; benchmark incomes associated with the Gini may still be estimated in the interior of a decile. This is due to the relationship (see formula in Table 1) linking the Gini to the position of the benchmark income in the income distribution.***

***I have added a discussion of these issues in lines 142-151.***

References

Niño‐Zarazúa, M., Roope, L. and Tarp, F., 2017. Global inequality: Relatively lower, absolutely higher. *Review of Income and Wealth*, *63*(4), pp.661-684.

Anand, S. and Segal, P., 2008. What do we know about global income inequality?. *Journal of Economic Literature*, *46*(1), pp.57-94.

3. Can the author provide the poverty line figures for the 10 countries parallel to the benchmark incomes? This can definitely show the policy-makers a clearer picture.

***Thank-you for this excellent suggestion. Based on the official poverty line and headcount poverty measure for each county, I have added a column to Table 2 indicating the percentile of the income distribution the official poverty line lies in. I agree that this presents a much clearer picture to policy-makers.***

***I have also added the following text in the Results section (lines 246-255).***

***“By way of comparison, Table 2 also contains estimates from the World Bank [10] and other sources ([11] for UK estimates; [12] for US estimates and [13] for Brazil estimates) of the poverty headcount ratio in 2010, based on the official poverty line – which is equivalent to the percentile of the income distribution in which the official poverty line lay. The benchmark incomes in all countries generally lay far above the official poverty line. The only exception is that the poverty line in South Africa, in the 53rd percentile, was slightly above the Absolute Gini’s 50th percentile benchmark – but well below the benchmark percentiles implied by all the other inequality measures. For example, across the ten countries, the benchmark percentiles implied by the Gini coefficient lay on average 50 percentiles above the official poverty line percentile (ranging from 32 to 61 percentiles).”***

4. It would benefit the readers if the author can discuss more about the role of correlation between inequality measures (I) and benchmark percentiles (p) in Table 3. I understand if the I-I, I-p, or p-p pairs are highly correlated, then the corresponding rankings are similar. But which one a government should use? As the benchmark income for AG is a constant and thus uncorrelated with all I and p, does it mean that it is not reliable and thus the government should not use it?

***Thank-you for this suggestion. In the revised manuscript I have added more discussion on this.***

***As the Gini coefficient is the only measure that is perfectly correlated with its benchmark percentile, this means that the Gini coefficient’s benchmark percentiles are consistent with the measure itself, in the sense that higher inequality necessarily means that subsidies can take place in higher percentiles before they become disequalizing – I think this is arguably an attractive property for an inequality measure. Conversely, the Absolute Gini being uncorrelated with its benchmark percentile may be considered unappealing.***

***While several of the inequality measures are not strongly correlated with each other, apart from the Absolute Gini, the different measures’ benchmark percentiles are all strongly correlated with each other. Thus, there is more agreement among the measures in where countries’ benchmark incomes lie than in how unequal the countries are. Moreover, as the Gini coefficient is strongly correlated with all the measures’ benchmark percentiles (apart from the Absolute Gini’s), ranking countries by benchmark incomes for any of these measures is very similar to simply ranking them by their Gini coefficient. Again, this is arguably an attractive property of the Gini coefficient.***

***I have expanded the discussion along these lines by adding new text (lines 204-207).***

5. Any explanation that USA’s wage inequality is the highest under AG and V measures? They triple S. Africa’s numbers but Gini’s measure shows the opposite. It would be better if the author can remind the readers when they use AG and V for analysis.

***Thank-you for this query and suggestion. As I have tried to make clearer in the revised paper (new paragraph added in lines 165-171), the reason for this is that, unlike relative inequality measures, absolute measures such as the Absolute Gini and the Variance are sensitive to the absolute gaps between incomes. As discussed by Niño‐Zarazúa et al. (2017), this means that in high-income countries absolute inequality is generally very high, even if relative inequality is very low, as the absolute gaps between incomes will generally still be high. Incomes in South Africa are much lower than in the USA. Thus, even though the relative gaps between incomes are extremely high in South Africa (much higher than in the USA), the absolute size of the gaps between incomes is much lower than in the USA.***

6. The conclusion part is a bit confused. It is said that “equalizing growth” cannot help reduce poverty, and that the incremental income between poverty line and benchmark incomes cannot help reduce poverty. What is the relationship between “equalizing growth” and “incremental income between poverty line and benchmark incomes”? Can you define “equalizing growth”? If a policy can increase income between poverty line and benchmark incomes, there is chance that the income below poverty line can also increase. It would be better if the author can cite some papers relating to “equalizing growth” policy that fails to ameliorate poverty.

***I have tried to make the conclusion clearer. It was certainly never my intention to suggest that “equalizing growth” cannot help reduce poverty!***

***The text that led to this misunderstanding was that “‘equalizing’ growth need not necessarily be poverty reducing. Incomes above the poverty line but below the benchmark incomes typically constitute a large proportion of a country’s income distribution. Growing these incomes can reduce inequality, even of the absolute kind, but not poverty.”***

***As the reviewer rightly suggests, I should have been much clearer here – thank you for pointing this out.***

***The point I was trying to make– and I feel this is an important point - is that it if, due to a particular pattern of economic growth, incomes increase primarily above the poverty line but below the benchmark income, inequality will fall but poverty will not be reduced. Thus, policy-makers should be careful not to assume that an increase in mean incomes, together with lower inequality (according to any of a wide range of measures), will necessarily mean that people have been lifted out of poverty. Instead, it is important to examine the overall pattern of growth, and in which percentiles of the economy it occurs.***

***I have rephrased the confusing ‘equalizing growth’ term in the last paragraph (and also in the Abstract). I have also substantially expanded the discussion in the last paragraph (lines 277-287) to explain this point more clearly.***

There are some typos in the paper:
1. Line 71: I_(MLD) instead of I_T

***Corrected – thank you.***

2. Line 109: “Iv is expressed in million (square)”?

***This text is actually correct. These numbers are expressed in millions. So, for example, for India I_V = 9,607,000.***

3. Line167: (57)th, (57)th instead of 56th, 56th as these are the thresholds for increasing inequality due to an increase in income, right?

***Thank-you. Actually the threshold lies two fifths of the way through the 56th percentile but I agree that just referring to the 57th percentile is cleaner and have done so in the revised paper.***
